# Multiphoton Microscopy for the Characterization of Cellular Behavior on Naturally Derived Polysaccharide Tissue Constructs With Irregular Surfaces for the Development of Platform Biomaterials

**DOI:** 10.3389/fbioe.2020.00802

**Published:** 2020-07-21

**Authors:** Mitchell Harling, Patrick Breeding, Travis Haysley, Mitchell Chesley, Michael Mason, Karissa Tilbury

**Affiliations:** ^1^Department of Chemical and Biomedical Engineering, University of Maine, Orono, ME, United States; ^2^Graduate School of Biomedical Science and Engineering, University of Maine, Orono, ME, United States

**Keywords:** biomaterials, biocompatibility, 2-photon microscopy, Second Harmonic Generation, cellulose

## Abstract

Over the past decade, the use of polymers as platform materials for biomedical applications including tissue engineering has been of rising interest. Recently, the use of naturally derived polysaccharides as 3-D scaffolds for tissue regeneration has shown promising material characteristics; however, due to complexities in composition, morphology, and optical properties, adequate spatial and temporal characterization of cellular behavior in these materials is lacking. Multiphoton microscopy has emerged as a viable tool for performing such quantification by permitting greater imaging depth while simultaneously minimizing un-favorable scattering and producing high-resolution optical cross sections for non-invasive analysis. Here we describe a method using endogenous contrast of cellulose nanofibers (CNF) using Second Harmonic Generation (SHG), combined with 2-photon fluorescence of Cell Tracker Orange for spatial and longitudinal imaging of cellular proliferation. Cell Tracker Orange is an ideal fluorophore to avoid the broad CNF autofluorescence allowing for segmentation of cells using a semi-automatic routine. Individual cells were identified using centroid locations for 3D cell proliferation. Overall, the methods presented are viable for investigation of cellular interactions with polysaccharide candidate biomaterials.

## Introduction

Over the past decade, interest in polysaccharide-based biomaterials for biomedical applications has dramatically increased. Common polysaccharide polymers used in biomedicine today include alginates, starches, chitosan, and cellulose nanofibers (CNF); all abundant, naturally sourced materials (Wahab and Saiful, 2016). These materials are highly attractive candidates for biomedical applications as they are readily modifiable using surface chemistry functionalization for mechanical, optical, and biocompatibility properties. Realization of these desirable and adaptable properties has led to the broad utilization in applications such as drug delivery, wound healing, and tissue engineering (Bacakova et al., 2004; Manian et al., 2008; Lin and Dufresne, 2014; Karadzic et al., 2015).

Tissue engineering applications of these materials include biomedical implants, three-dimensional cell culture systems, wound healing, and tissue regeneration scaffolds. Preparation of such materials requires an intricate balance of desired mechanical and biocompatibility properties. For example, an implantable polysaccharide-based medical device for bone regeneration requires tensile properties similar to that of the native bone, accompanied with trabeculae-like structures for cellular migration and cellular communication to activate osteoinduction, osteoconduction and osseointegration (Mieszawska and Kaplan, 2010; Bacakova et al., 2014; Tiwari et al., 2018). Overall, the high tunability of polysaccharides make them attractive candidate materials for regenerative tissue applications because of the capability to allow for growth of viable cells, adhesion, proliferation, migration, and maturation into integrated tissue networks (Loh and Choong, 2013). In order to vet a material’s ability to facilitate or induce such biological processes, it is necessary to quantify such attributes of the material’s interaction with biology at the cell–material interface.

The biocompatibility of a novel biomaterial that is intended to be used as a tissue engineering scaffold, such as CNF, is dependent on (1) the degree to which cells can infiltrate the material, (2) the biocompatibility of any degradation products, and (3) the ability of the material to serve as an artificial extracellular matrix (ECM) to guide cellular behavior (Williams, 2008). An early indication of biocompatibility is cell proliferation assessed using wide-field and confocal microscopy approaches, plate reader assays, and flow cytometry. Microscopy approaches are advantageous over plate reader assays and flow cytometry as they offer spatial information; however, wide-field and confocal microscopy have limited depth penetration, which is particularly troublesome in highly scattering biomaterials such as polysaccharides (Sheppard and Cogswell, 1990; Nozari et al., 2012; Simão et al., 2015; Dhaliwal et al., 2016). Flow cytometry approaches are quick and commonly used to differentiate cellular populations via the presence of surface receptors; however, cells must be removed from the candidate biomaterial either through mechanical scrapping or chemical digestion, potentially altering the cells (Karadzic et al., 2015; Ong et al., 2017). Atomic Force Microscopy (AFM) provides high resolution images of surface topological features suitable for studying fibril orientation and geometry, and the ability to derive mechanical properties including Young’s Modulus and viscoelasticity. Depth penetration is an issue for AFM as well, therefore, 3D biological interactions within complex candidate biomaterials such as polysaccharides are difficult to obtain (Variola, 2015; Iturri and Toca-Herrera, 2017; Marrese et al., 2017; Stylianou et al., 2018).

Multiphoton approaches to examine the interaction between cells and candidate biomaterials in 3D is an exciting alternative to conventional tools. For example, Second Harmonic Generation (SHG) imaging has been utilized as a tool to study fine features of CNF, as well as, phenomena at the cell-material interface (Cox et al., 2005). Second Harmonic Generation is a coherent, label-free process that occurs when 2 photons of identical energy interact with a non-centrosymmetric structure resulting in a single photon at half the wavelength. Common biological molecules that are SHG active include: fibrillar collagens, myosin, and cellulose (Nadiarnykh et al., 2007; Chen et al., 2012). Second Harmonic Generation can be coupled with other multiphoton modalities to create multimodal systems as well. Simultaneous SHG with Coherent Anti-Stokes Raman Scattering (CARS) microscopy has been used as a method for obtaining good specific contrast to examine the proliferation and adhesion of PC12 cells on CNF for neuronal growth (Jonsson et al., 2015). Previously, SHG imaging of Valonia and Acetobater cellulose identified characteristics of the depth dependence SHG directionality and polarization profiles to understand structural properties of the cellulose (Nadiarnykh et al., 2007). Similar work demonstrated the SHG signal dependence on fiber orientation and excitation beam polarization by probing spectral characteristics of dry versus hydrated CNF with Raman spectroscopy and SHG imaging (Zimmerley et al., 2010). Second Harmonic Generation coupled with Two-Photon Excitation Fluorescence (TPEF) imaging is another multimodal approach that shows promise as a viable multimodal platform to simultaneously image cellular features and morphology of the candidate polysaccharide biomaterial with minimal scattering and increased depth penetration.

Here, we present a method utilizing TPEF and SHG imaging to monitor cellular proliferation on CNF scaffolds. We first characterized the fluorescence of the CNF using Diffuse Reflectance Spectroscopy (DRS) and Steady State Luminescence Spectroscopy (SSLS), which illustrated its broad emission, leading us to explore photobleaching to optimize our contrast-to-noise ratio (CNR) for robust cellular imaging using common fluorophores. Using SHG microscopy; we confirmed that there was no structural alterations to the CNF structure post-photobleaching; however, the autofluorescence recovered within 24 h. The return of CNF autofluorescence within 24 h impinged on our ability to use a broad range of fluorophores or cellular NAD(P)H and FAD autofluorescence for 48–72 h studies. To avoid the impact of CNF autofluorescence, cells were stained with Cell Tracker Orange (CTO) to identify the cytoplasm of cells for up to 72-h for cell proliferation. A semi-automatic image algorithm was developed to segment cells from the background CNF autofluorescence. Additionally, the CNF organization was imaged using SHG microscopy and both signals were merged for 3D investigation.

## Methods

### CNF Film Preparation

Roughly 3 mm thick CNF films were prepared from a bulk source of 3 wt% CNF slurry (University of Maine Nanomaterial Pilot Plant, Process Development Center, Orono, ME) by confining 25 mL increments of slurry between two macro-porous ceramic interfaces and transferring into an 80°C oven for 24 h, producing bulk dried films that are approximately 250 μm. Thin 20 mm diameter circular films were then created from these bulk materials using a circular biopsy punch and were subsequently sterilized using a dry autoclave at 121°C for 60 min. The CNF films are mainly fibrous; pores are irregularly defined and are smaller than 10 μm. Moreover, the surface of the CNF films are irregular with high surface roughness facilitating pseudo-3D biomaterial interfaces. See Supplementary Figure S1 for a representative edge-on SEM image of CNF film. For cell culture experiments, films were seeded with MC 3T3 E1 (ATCC CRL-2593), mouse pre-osteoblasts, at a density of 100,000 cells/mL, suspended in 2 mL of complete media. Complete media consisted of MEM Alpha 1X (Gibco, Life Technologies) with 10% FBS (Gibco, Life Technologies) and 1% penicillin/streptomycin (Gibco, Life Technologies).

### Diffuse Reflectance Spectroscopy

Cellulose nanofibers DRS spectra were collected at 298 K, using a Mikropack DH-2000 deuterium and halogen light sources and an Ocean Optics USB4000 spectrometer (Ocean Optics, Inc.). Spectra were referenced with MgSO_4_. Data was processed using SpectraSuite and further analyzed using Origin.

### Steady-State Luminescence Spectroscopy

Cellulose nanofibers SSLS scans were collected at 298 K, using a Model Quantamaster-1046 photoluminescence spectrophotometer from Photon Technology International (Birmingham, NJ) using a 75 W xenon arc lamp combined with two excitation monochromators and one emission monochromator. A photomultiplier tube at 800 V was used as the emission detector. Cellulose nanofiber films were mounted on a copper plate using non-emitting copper-dust high vacuum grease. Excitation and emission scans were iteratively performed to identify peak CNF excitation and emission characteristics.

### Two-Photon Excitation Fluorescence Imaging

The custom-built two-photon microscope setup used consisted of a mode-locked Titanium Sapphire laser excitation source (Chameleon Ultra II; Coherent, Santa Clara, CA) directed into a laser scanning unit (FluoView 300; Olympus, Center Valley, PA) which was mounted onto an upright microscope stand (BX50WI; Olympus, Center Valley, PA). Laser power was modulated by an Electro-Optic Modulator (ConOptics, Danbury, CT), operated in a power range of 5–50 mW at the focal plane using a LUMPlanFLN 40x 0.8NA (Olympus, Center Valley, PA) water immersion objective. Circular polarization was used for CNF SHG imaging, verified at the focal plane by rotating a polarizer and experiencing no change in laser power.

Cells were labeled with CTO CMTMR dye (Thermo Fisher Scientific, Waltham, MA), and excited at 815 nm, with epi-fluorescence emission collected at 565 nm using a 582/64 nm bandpass filter (Semrock, Rochester, NY). The CNF fiber structure was imaged using SHG with a laser incidence wavelength of 890 nm and backward SHG collection at 445 nm using a 448/20 nm bandpass filter (Semrock, Rochester, NY). Both CTO fluorescence and backwards SHG signals were detected using a H7422 GaAsP PMT (Hamamatsu, Hamamatsu City, Japan). All 3D images were acquired using 3× optical zoom (85 μm field of view), 512 by 512 pixels, 1 μm step size, laser scanning speed of 2.71s/frame, Kalman 4 averaging without adapative modulation of laser power with increased imaging depth. The boundaries of the imaging stacks were determined based on the presence of SHG signal from the cellulose. Single optical stacks ranged between 40 and 70 μm in depth. Note this is much less than the actual thickness (250 μm) of the CNF films, due to the highly scattering nature of the CNF films. Cellular proliferation was monitored on three independent CNF films for each time point. Three areas of a single CNF film per time point were imaged using a grid-based selection process at 24, 48, and 72 h time points for a total number of nine independent imaging stacks per time point. The CNF films were only used at a single time point.

### Cellular Proliferation Image Analysis

**FIGURE 1:**
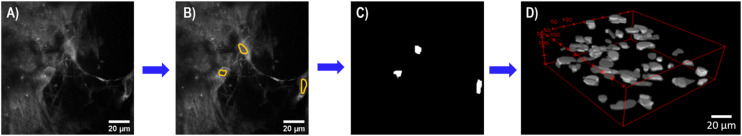
Flowchart summary of semi-automated cell analysis. **(A)** Representative single optical section of pre-osteoblasts with CNF autofluorescence. **(B)** Cell nuclei (yellow) were manually identified in every single optical section using ImageJ. **(C)** Segmented cell nuclei converted into binary mask for each optical section. **(D)** Representative 3D optical imaging stack of binarized cell nuclei used by ImageJ 3D Objects Counter to identify 3D centroid coordinates for each cell for cell counting. Scale bar is 20 μm. All images acquired using a 40× objective, 3× optical zoom at 815 nm at 5–7 mW.

Three-dimensional imaging stacks of CTO-labeled MC 3T3 E1 pre-osteoblasts fibroblasts were collected at 24, 48, and 72-h time points. Using a semi-automated process in ImageJ, cellular proliferation was quantified. Due to significant CNF autofluorescence, the ability to accurately segment the CTO-labeled cell cytoplasm was limited. The lack of contrast of cell nuclei was used to identify cells, similar to cell identification in optical metabolic redox imaging studies (Walsh and Skala, 2014). Cell nuclei in every optical section were manually segmented and converted into a binary mask these binary masks remained in the form of *z*-stacks, conserving the true *z*-coordinate of each cell. Using the ImageJ function “3D Objects Counter,” the 3D centroid coordinates for each cell, in every stack was determined as previously described by Bolte and Cordelieres (2006), to provide a robust metric of cellular proliferation (Figure 1).

## Results and Discussion

### Characterization of CNF Autofluorescence

**FIGURE 2:**
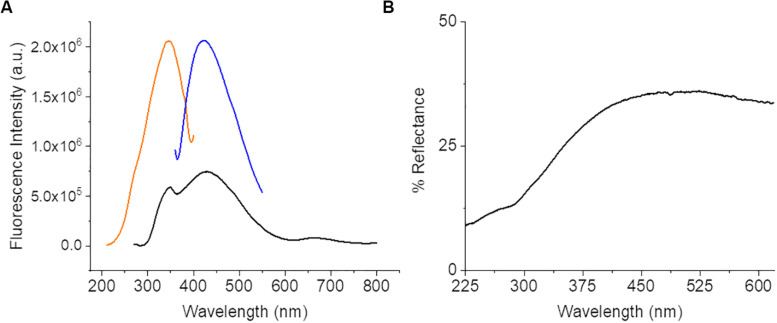
Steady state luminescence data from bare CNF. **(A)** Spectra displays a strong excitation peak at 320 nm (orange) and emission peak at 435 nm (blue) and a small intensity of emission present across a broad range from 370 to 720 nm (black). **(B)** CNF Diffuse Reflectance Spectroscopy data. Spectra collected of bare CNF.

The diffuse reflectance spectra (Figure 2) of CNF was about 40% or less across the entire spectrum. This could indicate a potentially very broad absorption since over half the light entering the system is not being reflected. The potential absorption bands of the CNF were further characterized using steady state luminescence with excitation scans at 270 and 420 nm and an emission scan at 420 nm. An absorption band was observed between 300 and 390 nm with a peak at 320 nm with a corresponding emission band of 395–490 nm with a peak emission at 420 nm. An excitation sweep was also performed at 270 nm and identified the predominant emission at 420 nm and a small but broad emission from 630 to 720 nm with a peak around 662 nm. Although there is fluorescence above 520 nm, the intensity is relatively weak (∼10× less than the 420 nm peak); therefore fluorophores with emission above 520 nm are likely suitable for optimizing cellular imaging on CNF films to minimize interference by CNF autofluorescence.

### CNF Photobleaching

**FIGURE 3:**
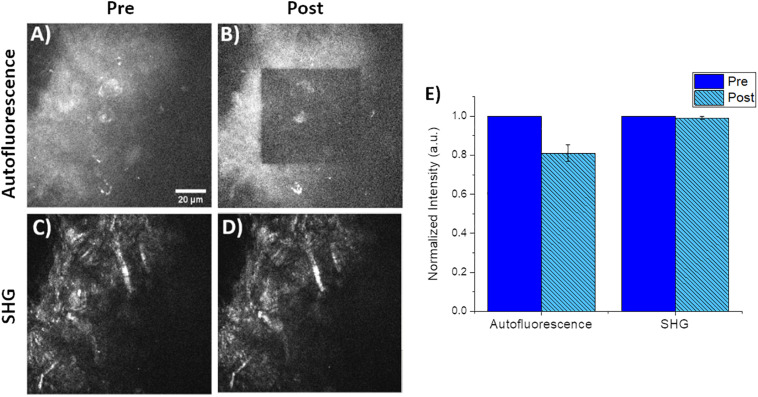
Representative pre/post photobleaching of CNF films. **(A,B)** Qualitative pre/post characterization of photobleaching of CNF films. Both images acquired using 760 nm excitation and fluorescence collected via a 582/64 nm filter and 3x optical zoom. Note the inner 42.5 μm by 42.5 μm square was the photobleached using 760 nm at 49 mW for 55 s with the same 40× imaging objective and 6× optical zoom. **(C,D)** Corresponding SHG images of the identical pre/post photobleached CNF film using 890 nm excitation and 448/20 nm for SHG collection. **(E)** Plot of normalized autofluorescence and SHG intensity of (*n* = 5) CNF films pre/post photobleaching, error bars represent standard deviation. Scale bar is 20 μm. All images acquired using 40× objective and 3× optical zoom with incident laser power ranging from 5 to 50 mW.

Small square regions (42.5 μm × 42.5 μm) within the original 85 μm field of view were photobleached using 760 nm light at 49 mW for a photon flux of 9.9 × 10^16^ photons/μm^2^⋅s for approximately 55 s. The choice of 760 nm as the photobleaching wavelength was approximately 2× the peak of 1-photon absorbance. We should note that the exact 2-photon absorption band is unknown for CNF and is likely highly variable depending on the tree species as well as processing steps and may be a topic of future publications for refinement of photobleaching processes. Photobleaching was confirmed by both visual and quantitative inspection of the decrease in autofluorescence intensity of the small photobleached area in images of the CNF film. Images were obtained immediately before and after photobleaching. A representative collection of pre/post images are seen in Figures 3A–D; where A and B are the CNF fluorescence and C and D are SHG, respectively. The average intensity within the photobleached ROI was compared to the pre-photobleached ROI where the average intensities were normalized to pre-photobleached ROI average intensity values. Comparison of the average Pre and Post-photobleached SHG intensities was not different; however, the autofluorescence intensity decreased by 20% after photobleaching (Figure 3E). The lack of intensity change in the SHG images collected with the 448/20 nm bandpass filter demonstrates that the dominant signal in this region is SHG; with minimal CNF autofluorescence detection. This is a promising indicator that SHG is a highly sensitive approach for label-free characterization of CNF fibers. Previous work has used SHG to validate the lack of structural damage resulting from photobleaching (Shahin et al., 2014). Visual inspection of the pre/post photobleached SHG images demonstrated no loss of structure.

**FIGURE 4:**
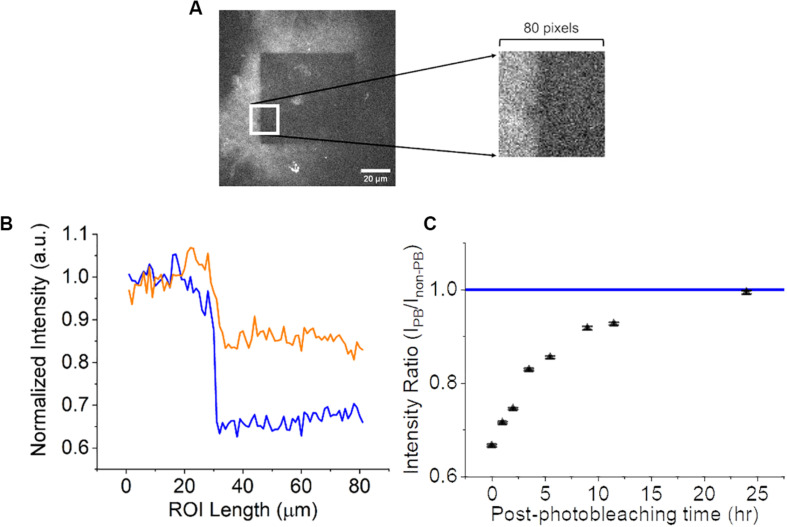
Analysis of CNF fluorescence recovery over 24 h. **(A)** 80 by 80-pixel ROI (white square) of CNF with and without photobleaching. Left portion of ROI is not photobleached and the right portion of ROI is photobleached. **(B)** Normalized intensity as a function of × position along the line profile at 0-h (blue) and 5.5-h (orange) within the ROI. **(C)** Scatter plot (*n* = 3, error bars = standard deviation) of the ratio of photobleached/non-photobleached CNF intensity as a function of time approaching the normalized native CNF autofluorescence (blue line at 1). Scale bar is 20 μm.

Once photobleaching was confirmed, a longitudinal time-lapse study was performed to examine if the photobleaching effects were permanent or if there was a recovery period. For this experiment, a CNF film was photobleached and the autofluorescence was measured as previously described at the following time-points: 0, 1, 2, 3.5. 5.5 9.5, 11.5, and 24 h. CNF films were left at 25°C room temperature and 45% humidity during the length of the experiment on the microscope. A material topological feature distinctly visible in images was referenced as a fiduciary marker on the CNF to ensure that the same focal plane was imaged at all time-points. A single square ROI as seen in Figure 4A (80 pixels by 80 pixels) consistent across each time point image was selected to include the non-photobleached CNF region, photobleached region and edge between the two. The ROI was ‘vertically condensed’ by summing the raw pixel intensity values in each column (80 pixels). The intensity profiles of the vertically condensed ROI were self-normalized to the non-photobleached regions within the ROI for each time point. Representative intensity profiles 0-h (blue) and 5.5 h (orange) are shown in Figure 4B. To investigate the extent of autofluorescence recovery, the ratio of the average normalized intensity of the non-photobleached and photobleached were calculated and compared to the normalized intensity of non-photobleached CNF which had a value of 1 (Figure 4C). The fluorescence recovery at 24-h approached the nascent CNF fluorescence intensity clearly limiting the viability of photobleaching techniques for time-course imaging studies.

The specific mechanism of this fluorescence recovery is difficult to determine, as there are numerous factors. In general, it is well known that intense illumination causes photobleaching of the fluorescent molecules within the excited area, which in turn creates a diffusion gradient that causes an inflow of fluorophores from the surrounding area into the photobleached region. The diffusion of these fluorophores has been a well-studied field, where models are applied to represent the mechanism of diffusion. These models accurately predict experimental measurements when parameters are known such as: (1) arrangement of the fluorophores, (2) structural hierarchy and heterogeneity of the fluorescent and non-fluorescent species, and (3) polydispersity of the fluorescent species (Sullivan et al., 2015). As many of these properties of CNF are unknown, it is difficult to model fluorescence recovery. A plausible explanation for the source of CNF autofluorescence and its recovery is the interaction of the CNF backbone with water molecules. During photobleaching, water molecules are likely driven outside the ROI, but will slowly diffuse back into the region, returning to its native autofluorescent state.

The photobleaching recovery results coupled with the broad autofluorescence profile of CNF make obtaining clear images of cells within the CNF scaffold difficult. With both of these pieces of information, however, we can utilize exogenous labels to capture cells clearly and distinctly. The benefit in utilizing these labels in this scenario is their tightly controlled synthesis allows for narrow excitation and emission peaks, meaning even with the broad fluorescence profile of CNF, capturing cellular fluorescence can be done. CTO was chosen as the ideal candidate for this study because of its peak emission at 565nm is longer than the strong CNF autofluorescence band between 430 and 500 nm (Figure 1A) to permit better contrast separation between the cells and CNF background for segmentation.

### Cellular Proliferation

**FIGURE 5:**
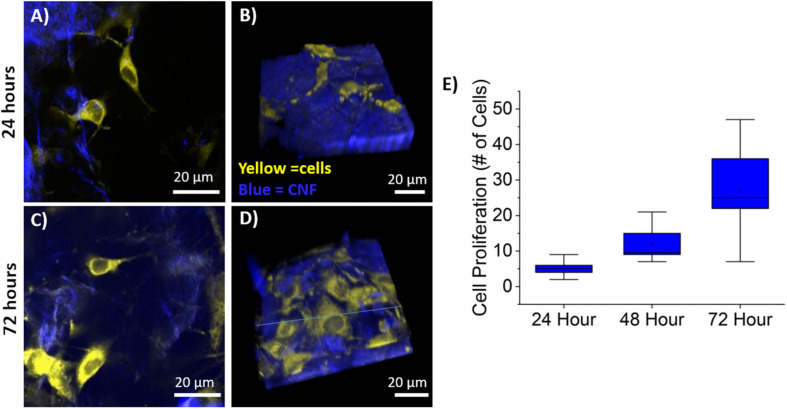
Cell Proliferation on CNF. **(A,C)**, Representative single optical sections of CNF films at 24 **(A)** and 72 **(C)** with CTO labeled pre-osteoblasts in yellow and CNF SHG signal in blue, false color. **(B,D)** Representative 3D rendering of SHG (blue) and CTO labeled pre-osteoblast imaging stacks at 24 **(B)** and 72 **(D)** hours. Imaging stack depth ranged from 40 to 70 μm with 1 μm step size. **(E)** Box and whisker plot of cell proliferation of pre-osteoblasts at 24, 48, and 72 h on CNF films (*n* = 9 for 24, 72 h, *n* = 6 for 48 h) quantified via semi-automated analysis. Scale bar is 20 μm. All images acquired using 40× objective and 3× optical zoom with incident laser power ranging from 5 to 50 mW.

Cellular proliferation was quantified via the identification of cell nuclei within the 3D image stack using the previously described semi-automated segmentation algorithm. Average cell counts from each time point are shown in Figure 5 where the line is the median and the top and bottom of the box plots represent Q1 and Q3. Sample sizes of *n* = 9 (imaging areas) were used for 24 and 72-h time points, while *n* = 6 was used for 48-h. The MC 3T3 E1 pre-osteoblast mouse cells proliferated as expected with statistically significant growth between each time-point as determined by a Wilcoxon rank sum test with *p*-values of 0.0053, 0.0012, and 0.059, respectively.

This semi-automated segmentation algorithm image analysis routine demonstrates a straight-forward approach to examine longitudinal cell proliferation as an initial biocompatibility screening assay for candidate polysaccharide-based biomaterials. Furthermore, this segmentation algorithm is flexible (i.e. introduction of filtering, thresholding, and rescaling) and offers a robust tool for more complex analysis of biological interaction with polysaccharides. For example, using both the 3D centroid location of the cell coupled with the SHG signal of the CNF may allow for more specific characterization of cellular interaction with complex biomaterial topology and its impact on cellular penetration and migration. Additionally, the organization and morphology of individual cells within the polysaccharide can be studied. Finally, using this method in conjunction with other labels or different cellular morphological characteristics opens the door to a wide variety of applications for examining the cell–material interface including cell signaling events.

## Conclusion

With the rising interest in using polysaccharide polymers as biomaterials, materials with irregular surface topologies, the development of novel methods to non-invasively monitor the pseudo-3D growth and maturation of cells with candidate biomaterials is needed. Beyond irregular surface topologies, candidate polysaccharide polymers frequently exhibit broad autofluorescence. In this work, we explored photobleaching as a potential approach to minimize the impact of CNF autofluorescnece. Photobleaching successfully diminished the autofluorescnece of the CNF without damaging the CNF structure; however, the return of autofluorescence within 24 h limits its utility for CNF specifically. The recovery of autofluorescence is likely specific to each polysaccharide polymer; therefore the photobleaching methodology presented is broadly applicable in the development of imaging assays of new candidate biomaterials. Despite the return of autofluorescence, a viable window in CNF’s optical properties displaying low amounts of autofluorescence from 550 to 600 nm was observed and taken advantage of to utilize CTO. Combining this with SHG proved to be a viable method to provide useful, quantitative information on longitudinal cell proliferation on the CNF scaffold in a three-dimensional manner. These 3D image stacks were analyzed by our semi-automated image analysis method that demonstrated statistically significant cell proliferation over the 72-h period. The CNF films used in the experiments were very dense with small pores which likely prevented cellular in-growth. Initially, it may be counter-intuitive to use these CNF films; however, this type of CNF film highlights the challenges of polysaccharide biomaterials. Many polysaccharide materials possess ill-defined autofluorescence properties and have poorly controlled surface properties. Therefore, the CNF films used in this study were selected to highlight the specific opportunity afforded by 2-photon microscopy techniques and its ability to section through strongly autofluorescent and scattering media to identify cells. In the present study, cells were found as deep as 30 μm from the CNF film surface within a single field of view where the superficial surface of the film was based on the presence of any SHG signal in the entire field of view. It should be noted that the presence of cells within 30 μm from the superficial surface is not an indicator of cell penetration as there is no evidence of CNF material surrounding the cells as one would expect if the cell penetrated and remodeled the CNF film. Furthermore, the pre-osteoblasts used in this study do not possess appropriate cellular machinery to modify the CNF films, nor were the CNF films engineered to be degradable. The presence of cells within different axial planes is most likely due to the surface roughness of the CNF films. Using typical wide-field fluorescence techniques, only cells on the most superficial surface would be resolvable. Furthermore, use of confocal imaging approaches presents limitations in terms of fluorophore selection to avoid the CNF autofluorescnce and decreased depth penetration due to increased scattering at shorter wavelengths. In summary, the 2-photon based imaging processes demonstrates the ability to optical section through CNF films while allowing identification of cells and the matrix.

Overall, the use of a multiphoton approach to probe the cell-material interface has proven to be more effective than conventional tools, although, our method is not without its set of limitations. Light penetration into the CNF without significant scattering still limits our capabilities to be able to probe the cell-material interface deeper than a thin film. The use of longer excitation wavelength ranges may further reduce scattering and improve depth penetration. The limited image penetration means that although we can obtain good contrast images on the CNF films used, optical clearing techniques will be required for more sophisticated cell/biomaterial interactions. With these physical limitations in mind, however, our straightforward image analysis relying on identification of fluorescently labeled objects lends itself to further applications using other exogenous fluorophores. Currently, we are also exploring the capability of trainable segmentation tools such as the Weka Segmentation tool in ImageJ to minimize user-input for more robust image analysis. As more robust image analysis tools are under development, the ability to monitor cellular morphology feasures will promote longer-term biocompatibility studies using transfected cell-lines for long-term tracking of cellular interaction with the biomaterial. The wealth of information that can be gleamed about cell–material interactions from a cell’s morphology in a biomaterial and the wide availability of labels for probing more specific cellular features motivates continued development and use of advanced microscopy and computational methods.

## Data Availability Statement

The datasets generated for this study are available on request to the corresponding author.

## Author Contributions

MH, PB, TH, MM, and KT contribued to the conception and design of the study. MH, PB, and TH performed all the experiments. MC made all the CNF samples and performed all the SEM imaging. MH, PB, MM, and KT wrote the manuscript. All authors contributed to manuscript revision, read and approved the submitted version.

## Conflict of Interest

The authors declare that the research was conducted in the absence of any commercial or financial relationships that could be construed as a potential conflict of interest.

## Supplementary Material

FIGURE S1 Edge-on cross-sectional SEM image of CNF film torn through tension. Imaging performed using Zeiss Nvision 40-38-14 with a 1.0 KX magnification, EHT = 3.0 kV and 1.00 μ Torr vacuum. The structure is primarily fibrous with small pores irregularly dispersed and the surface is rough seen in upper left corner. Scale bar is 10 μm.
