# Supplementary figures and images for: Multiphoton Microscopy for the Characterization of Cellular Behavior on Naturally Derived Polysaccharide Tissue Constructs With Irregular Surfaces for the Development of Platform Biomaterials
Source: Front Bioeng Biotechnol. 2020 Jul 21;8:802. doi: 10.3389/fbioe.2020.00802 (PMC7396702; doi:10.3389/fbioe.2020.00802)

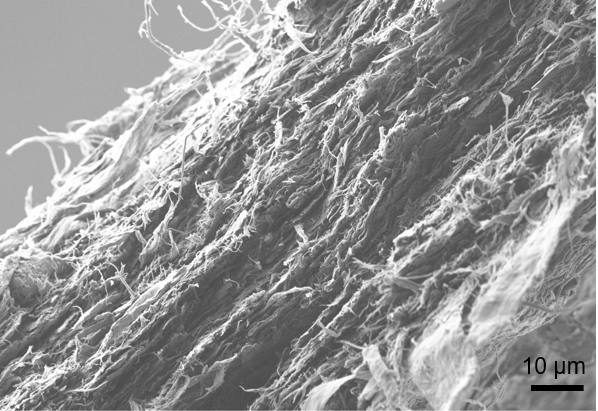

Supplement: FIGURE S1 — Edge-on cross-sectional SEM image of CNF film torn through tension. Imaging performed using Zeiss Nvision 40-38-14 with a 1.0 KX magnification, EHT = 3.0 kV and 1.00 μ Torr vacuum. The structure is primarily fibrous with small pores irregularly dispersed and the surface is rough seen in upper left corner. Scale bar is 10 μm. [file Image_1.TIF]
